# Effectiveness of checklist-based box system intervention (CBBSI) versus routine care on improving utilization of antenatal care visits in Northwest Ethiopia: a cluster randomized controlled trial

**DOI:** 10.1186/s12913-022-07894-7

**Published:** 2022-04-09

**Authors:** Netsanet Belete Andargie, Gurmesa Tura Debelew

**Affiliations:** 1 Ethiopian Ministry of Health, Addis Ababa, Ethiopia; 2 Department of Population and Family Health, Jimma University, Jimma, Ethiopia

**Keywords:** Fourth antenatal care, Cluster randomized controlled trial, Northwest Ethiopia

## Abstract

**Background:**

In Ethiopia, the proportion of mothers who attend the fourth antenatal care visit is lower than the proportion who attend the first visit. Although the reasons for these dropouts were investigated, few studies introduced interventions to promote the fourth antenatal care visit. Hence, the aim of this study was to assess the effectiveness of checklist-based box system intervention on improving fourth antenatal care visit.

**Method:**

This study employed a double-blind, parallel-group, two-arm cluster randomized controlled trial to compare the effectiveness of checklist-based box system intervention with the usual standard of care as a control arm. Study clusters are assigned to intervention and control arm in 1:1 allocation ratio using simple randomization technique. Pregnant mothers below 16 weeks of gestation were enrolled. Open data kit was used to collect data from the baseline and end-line surveys, and STATA version 15.0 was used to analyse the data. A difference-in-difference estimator was used to compare fourth antenatal care visit between the intervention and control groups across time. Mixed effect multi-level logistic regression was used to examine the relationship between the dependent and independent variables.

**Result:**

Data were collected from 2224 mothers who belong to 15 intervention and 15 control clusters. The difference in difference estimation resulted in a significant difference (26.1, 95%CI: 18–34%, *p < 0.0001*) between the intervention and control groups. Similarly, as compared to controls, the fourth antenatal care visit was found significantly higher in the intervention clusters (432 (85.2%) Vs. 297 (53.7%), *p < 0.0001*)/(AOR:5.69, 95% CI:4.14–7.82). Mothers who were knowledgeable about the services given during antenatal care visits (AOR: 2.31, 95% CI:1.65–3.24) and mothers who had a high level of social support (AOR:1.47, 95% CI: 1.06–2.04) were more likely to attend the fourth antenatal care visit.

**Conclusion:**

Implementation of checklist-based box system intervention resulted in a statistically significant effect in attendance of fourth antenatal care visit. Community-level variables were found to be more important in explaining variability in the fourth antenatal care visit. It is recommended that the intervention be implemented on a larger scale.

**Trial registration:**

ClinicalTrials.gov, Retrospectively registered on 26/03/2019, with trial registration number-NCT03891030.

## Background

The concept of continued utilization of maternal health care services has been identified as critical in combating maternal and new-born mortality [1]. In developing countries, however, a lack of/underutilization of key maternal health services such as antenatal care (ANC), skilled delivery, and postnatal care (PNC) has contributed to a high rate of maternal mortality [2].

One explanation for poor health outcomes among Ethiopian women is that a large proportion of women do not fully utilize modern health care services [3]. The last three consecutive demographic and health surveys (DHS) conducted by the Ethiopian government (EDHS 2014, 2016, and 2019) revealed that women were more likely to attend the first ANC visit than the fourth ANC visit [4–6]. This is also evidenced by low coverage of tetanus toxoid (TT) vaccine uptake, syphilis screening, use of insecticide-treated bed-nets, and suboptimal uptake of HIV prevention of mother-to-child transmission (PMTCT) services by pregnant women [7].

According to the literature, maternal health follow-ups build on each other; mothers who completed their fourth ANC visit were more likely to deliver at health facilities, and mothers who delivered at health facilities had a higher chance of receiving postnatal care services [8].

Multifaceted factors influence the use and non-use of maternal health services in general and ANC in particular. Evidence suggests that a mother’s education and exposure to trusted health information sources influence ANC utilization [9]. ANC knowledge, which is influenced by exposure to health information sources, has been identified as influencing ANC initiation within the recommended time frame [10] and utilizing consecutive ANC visits [11]. Furthermore, sociological concepts such as social support for women influenced women’s participation in prenatal and continued ANC utilization [12, 13]. Works of literature from Ethiopia investigated the reasons for not achieving the fourth ANC visit [14–16]. However, there are few interventional studies aimed at improving the utilization of the fourth ANC visit.

Because ANC is an important entry point for other maternal health services, a checklist-based box system intervention was designed and implemented with a two-pronged approach of demand creation and dropout tracing mechanisms. This intervention aimed to contribute to the larger gap between ANC one and four visit utilizations. This novel approach introduced mechanisms that aid in reminding health professionals about mothers who were failing to attend recommended ANC visits and demand creation activities to encourage women to return to the service. Because this demand creation was intended to be delivered at the community-level, cluster randomization was used to avoid information contamination.

The primary health care units of Ethiopia’s health system, composed of primary hospitals, health centers and five satellite health posts, were used to implement the checklist-based box system intervention. Health posts are primarily staffed with health extension workers (HEWs), spending 75% of their time on home-to-home visits providing promotional and preventive interventions and 25% of their time at health posts [17]. Maternal health care services have been identified as a cost-effective and proven intervention for reducing maternal and neonatal morbidity and mortality. As a result, this study aims to compare the effectiveness of checklist-based box system intervention to routine care in improving attendance at the fourth ANC visit, which indirectly contributed for maternal and neonatal morbidity and mortality reduction.

## Method

The trial protocol for this study was published [18], and the trial was retrospectively registered on ClinicalTrials.gov, on 26/03/2019 with trial registration number, NCT03891030.

### Design

A two-arm, double-blind, parallel-group cluster randomized controlled trial was conducted to evaluate the effectiveness of checklist-based box system intervention on improving attendance of fourth ANC visit.

### Setting

This study was conducted in East Gojjam zone, one of the administrative zones of Amhara region, located in North-western Ethiopia. According to 2019 Ethiopian mini Demographic and Health Survey (M-EDHS), the region’s maternal health service utilization for the first and fourth ANC was 82.6 and 50.8%, respectively [6]. According to the 2016 EDHS, more than half of (54.1%) women aged 15–49 years in the region had no formal schooling. Similarly, the majority of (83.5%) women of reproductive age (WRA) did not use any of the three media outlets (newspaper, radio, and television) at least once a week [5].

### Sample size determination

The sample size for this study was calculated following a recommendation for cluster randomized controlled trials with an equal-sized and fixed number of clusters [19]. This study is part of a larger study that included ANC, skilled delivery, and PNC. From the three options, the proportion of PNC was chosen to provide the largest sample size. Assumptions: the third postnatal care in the control group was 16.0% [20], number of clusters available – 30 (15 per cluster on each arm), 95% confidence interval and 80% power, the intra-cluster correlation coefficient of 0.04849 [21]. The sample size was then calculated to determine the number of observations per cluster using a two-sample comparison of proportion with normal approximations. STATA version 13 was used to run the calculation. Assuming individual randomization, the sample size per arm was 194. When the cluster randomization was used, the average cluster size required was 40, for a total sample size of 1200 pregnant mothers (600 from intervention and 600 from control). As a result, each baseline and end-line survey included 1200 mothers. The minimum detectable difference was set to be 12%.

### Randomization and sampling procedures

Debere-markos, Gozamin, and Machakel were chosen from among the sixteen districts available in East Gojjam Zone on the bases of confirming that none of the three districts had received an intervention/project aimed at improving utilization of maternal health services.

The randomization units were health posts/Kebeles in selected districts. Individual randomization was not considered because a component of this trial (demand creation) was planned to be delivered at the community-level, which would result in information contamination. Following the completion of cluster recruitment, the study team used SPSS-generated random sequence to assign clusters to the intervention and control arms in a 1:1 allocation ratio.

### Participant eligibility criteria

Participants in this trial were pregnant mothers under 16 weeks of gestation who lived in the designated districts. However, mothers who required hospitalization due to severe clinical complications and mothers who required a different type of ANC follow-up than the recommended focused ANC were excluded from the study.

### Screening and enrolment

The screening and enrolment for this study were primarily handled by HEWs. HEWs are females who have completed the 10th grade. They are recruited from the community they serve and are deployed after a one-year formal training period. They are government employees who are primarily in charge of health promotion and prevention activities [22]. A family folder: a detailed record of household-level data at health posts was used to assist HEWs in locating WRA during a community-level survey. After being identified as WRA, mothers were subjected to a two-stage screening process before being enrolled in the study.

First, HEWs performed community-level screening using Stanback et al., [23] pregnancy screening checklist. This checklist was used to identify suspected pregnant women. Mothers under and on 16 weeks of gestation were targeted in this community survey. Suspected pregnant women were given a referral slip, which was then given to a nearby health center; the visiting HEWs kept a copy of the referral slip for further follow-up. Second, for laboratory confirmation of pregnancy, a facility-level screening using beta-human chorionic gonadotropin (HCG) urine test was performed. Mothers who had a confirmed pregnancy were enrolled in the study and received their first ANC on the same day.

### Blinding

The intervention was concealed from mothers and outcome assessors. Despite receiving all of the intervention’s listed packages, mothers were unaware that they were in a different treatment group and receiving a new intervention. Because this study used cluster randomization, mothers from the same catchment received the same intervention, making them unaware that they were taking part in a new intervention because those who needed the service were receiving it. In addition, the familiar HEWs provided the community-level interventions.

Data collectors were unaware of the intervention, the intervention was delivered and data was collected by a separate group of health workers. Although intervention providers were aware of the intervention, data collectors were unaware of which groups were receiving the intervention and which were not. Furthermore, they are oblivious to the reason for the intervention and the objectives it is meant to achieve.

### Intervention description

#### Trial arm I/intervention

**Fig. 1:**
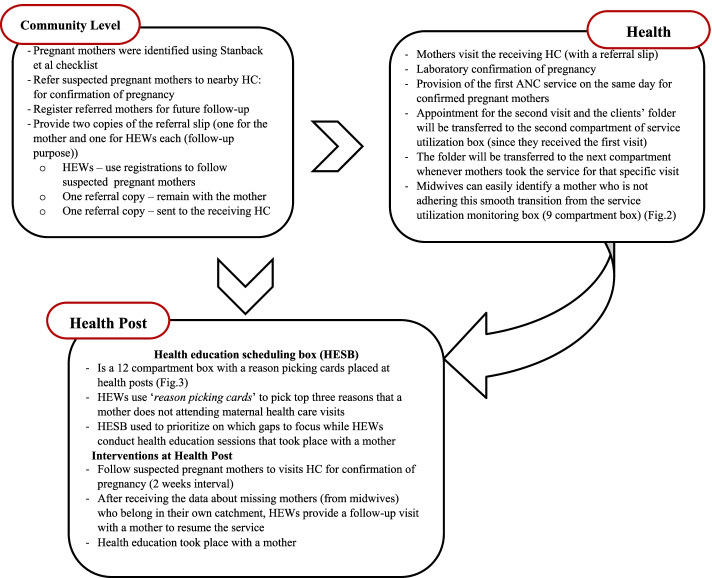
Summary of the intervention: at community, health post and health center level

To increase ANC service utilization, this trial used community-level demand creation and facility-level dropout tracing mechanisms (Fig. 1)*. *Community-level surveys were carried out for two reasons: first, to identify suspected pregnant mothers and refer them to a nearby health center; and second, to identify factors that prevented mothers from attending ANC follow-ups at health facilities.

**Fig. 2:**
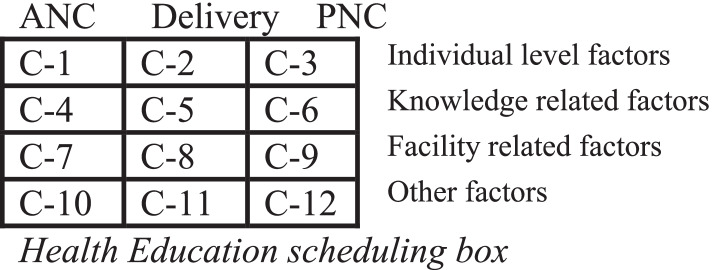
A 12-compartment health education scheduling box. For example, if a knowledge-related factor is raised as the most common reason for missing ANC visits, the reason picking card will be placed in C-4. Health education will be focused on compartments with more cards (C-Compartment, ANC- Antenatal care, PNC- Postnatal care)

The HEWs then identified the top three issues raised by mothers, documented using reason picking cards and provided problem-based health education. To make health education sessions more evidence-based, reason picking cards were used. The health education sessions held at the mother’s home were guided by addressing the most frequently mentioned reasons for maternal health service non-utilization. This data was gathered through community surveys, and each mother will receive at least three health education sessions throughout her pregnancy, delivery, and postnatal period. Mothers who did not attend any of the recommended visits, on the other hand, would receive additional health education in proportion to the number of visits missed (Fig. 2). A more detailed explanation about the intervention could be accessed from the study’s published protocol [18].

**Fig. 3:**
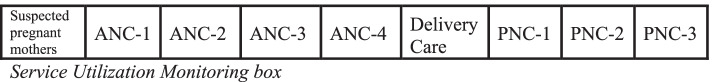
Service utilization monitoring box. The first compartment contains a copy of a suspected pregnant mother’s referral slip. The second compartment contains mothers who received the first ANC, the third compartment contains mothers who received the second ANC, and so on. A mother’s individual folder will be transferred to the second compartment if she received her first ANC visit. Missed mothers can be easily identified and communicated with during this process. (ANC stands for antenatal care, and PNC stands for postnatal care)

Once the mother was enrolled and received the first ANC at the health center level, the service utilization dropout monitoring box was used to track consecutive visits. Whenever the mother came to health facility and receive the service, her individual folder was moved to the next compartment across the health service monitoring box (Fig. 3)*.* The timing of ANC visits from the first to the fourth ANC followed the World Health Organization (WHO) focused ANC model (i.e. first ANC (before 16 weeks of gestation), second ANC (24–28 weeks), third ANC (30–32 weeks), and fourth ANC (36–40 weeks). Dropouts were communicated to HEWs where the mothers belong by the midwife tracing that dropout.

#### Trial arm II/control

Mothers in the control arm received the government’s existing routine care. In the routine maternal health service delivery, there was no community level screening (to improve early attendance at antenatal care) mothers visit health facilities for the first ANC based on their convenience, when mothers were appointed for a follow-up visit the mothers were given appointment cards for their next visit, there were no service utilization monitoring boxes to monitor service utilization dropouts, there was no knowledge gap assessment to be considered in health educations, the approach to awareness creation and health education mechanisms was in mass during pregnant mothers conference, and there was no individual based/person-based health education.

### Intervention process

A detailed intervention package outlining the steps to be taken while carrying out this trial was developed*. *Other supporting documents, such as training manuals, person-centered manuals for HEWs and manuals for health care providers (HCP), an orientation tool for local health administrators, and specifications for both boxes, were also developed. A sensitization workshop for local health administrators, and training for HEWs and HCP was held. The boxes were then purchased for health posts and health centers, and they were distributed along with the necessary number of printed checklists, referral slips, registers, and manuals. Then a community-level survey and enrollment process was initiated. On-site supervisions and follow-up visits were carried out based on the previously developed compliance parameter.

The trial was originally scheduled to last for ten months, however from the time the first mother with a confirmed pregnancy enrolled in the study and received the first ANC at the health center on January 3rd, 2019, to the end of follow-up for the last rounds of mothers on August 27th, 2020, it took around 20 months. This extended timeframe was due to the following challenges. The family folder at the health post level was not updated; additional efforts were made to locate WRA who were not on family planning. As HEWs delivered a component of this intervention, there were occasions such as annual leave, maternity leave, and transfer to other areas of the trained HEWs. This was a critical issue on health posts where there was only one HEW on staff. We used the existing weekly meeting platform between health centers and health posts for reporting suspected pregnant mothers (to send a copy of the referral slip from health post to health center). However, these meetings were not held regularly, especially for rural health facilities, and HEWs were sometimes absent from weekly meetings. Even though only a few participants experienced this, some mothers had their HCG tests rescheduled due to the test’s unavailability.

### Data collection tools and techniques

A structured and pre-tested questionnaire was used to collect data. The data collection questionnaire was developed after reviewing relevant literature, national [24], and international standards [25]. This trial used the PRECEED-PROCEED model as a lens [26]. This model emphasizes fundamental concepts such as health information sources and social forces as important environmental influences on health behaviour, personal desire variation may necessitate individualized care, and health behaviours that are unacceptable to society should be approached with caution [27, 28]. The English questionnaire was translated into Amharic (local language of the study area) for the data collectors. The Amharic version of the questionnaire has been uploaded to the kobo-tool box. Then, for the baseline and end-line studies, an open data kit (ODK), a software that uses mobile devices to collect and submit data to an online server, was used to conduct face-to-face interviews.

### Variables and measurement

The primary goal of this trial was to determine the effectiveness of checklist-based box system intervention on improving maternal health care utilization (ANC, skilled delivery and postnatal care). The findings in this study were focusing on the first outcome: improving attendance of the fourth ANC visit. As a result, the fourth ANC visit status was compared between the intervention and control clusters. Following that, additional factors influencing the use of the fourth ANC visit were investigated. For this analysis, ‘fourth ANC visit was identified as a dependent variable. Similarly, kebele was taken as a clustering variable, and then the independent variables were categorized as level 1 (individual-level variables) and level 2 (community and facility-level variables) variables. A more detailed list of variables with their description and measurement was included in Table 1.

**Table 1:** Description of study variables, East Gojjam Zone, Northwest Ethiopia, January 2019–September 2020

Variables	Description	Measurement
**Dependent Variable**		
Number of ANC follow-ups	Mother were asked how many ANC visits did they have for their last pregnancy	This was recorded as 0 for less than four visits and 1 for four ANC visits
**Independent Variables**		
**Level 1 Variables**		
**Individual level variables**		
Age	Age of the participant in completed years	A continuous variable and recoded in to three categories 15–19, 20–29 and 30–49
Level of education	The highest level of education that the mother attended	Categorized in to four groups: Non-formal education, Primary education (1–8), secondary education (9–12) and above 12 grade
Marital status	Marital status of respondents	Categorized in to four categories: Single, married, separated, widowed
Wealth quantile	Questions were adopted from EDHS and wealth index was computed using principal component analysis	Categorized in to five categories: Poorest, Poor, Medium, Rich and Richest
Parity	Total number of deliveries a mother had	Categorized in to three categories: one, 2–4 deliveries and ≥ 5 deliveries
Knowledge on ANC	Knowledge of the respondents on the services needed and provided during ANC: Do pregnant mother need ANC, Do pregnant mother need ANC regardless of illness, time to initiate ANC, minimum number of ANC visits, need for TT vaccination, How many TT vaccinations needed, the need for ion folate during pregnancy and the need for additional food during pregnancy.	A composite index of these eight variables was created and dichotomized using the mean score: Those who score the mean and above were categorized as *‘Knowledgeable’*, those who score below the mean ‘*Not knowledgeable’*
Knowledge on danger signs of Pregnancy	Knowledge of the respondents of danger signs of pregnancy: Vaginal bleeding, Sudden gush of fluid or leaking of fluid from vagina, dizziness and blurring of vision, Severe headache not relieved by simple analgesics, Sustained vomiting, swelling, loss of fetal movement, convulsion, Premature onset of contractions (before 37 weeks), Severe or unusual abdominal pain and chills/fever.	A composite index of these eleven variables was created and dichotomized using the mean score: Those who score the mean and above were categorized as *‘Knowledgeable’*, those who score below the mean ‘*Not knowledgeable’*
Pregnancy wontedness	Participants were asked whether their last pregnancy was wonted or not	Coded as ‘Yes’ or ‘No’
Gestational age at first ANC	Gestational age while mothers visited health facility for their first ANC visit	Recoded in to two categories as: ‘greater than three 16 weeks’ and less than or equal to 16 weeks
Compassionate and respectful care	A mother receiving MHC in a compassionate and respectful way: free of physical abuse, detention, non-confidential care, non-consented care, abandonment/neglect, and non-dignified care	Coded as ‘Yes’ or ‘No’
**Level 2 Variables**		
**Community Level**		
Place of residence	The place where the respondent usually belongs	Coded as Urban and rural
Average Distance	Approximate distance of participants home from nearby health facility in minutes/on foot	A continuous variable, recoded in 2 categories: 0–30 min as ‘0’ otherwise ‘1’
Influence of significant others in the process of receiving MHC	Participants were asked whether there is anyone who negatively influences them on the process of utilizing MHS at health facilities	Coded as ‘Yes’ or ‘No’
Social Support	Fourteen elements of SS questions: gets visits from significant others, getting useful advises, discussion on problems, having care at the time of labor and delivery, feeling loved, others thankful on them, getting help on household chores, help with money at emergency, help in transportation, card when sick, attending community level discussions, member of any religious cast, attending public meetings and help in case of conflicts	A principal component analysis was conducted, and a composite index was created using the principal components, and this was dichotomized using the mean score: those who score the mean and above were categorized as having *‘Good social support’* and otherwise *‘Poor social support’*
Facility Level		
Receiving maternal health care free of charge	Respondents were asked about their facility MHC visit and the payment associated with it	Participants who always got the service free of charge coded as ‘1’, most of the time as ‘2’, and never ‘3’
**CBBSI Intervention**	Kebele/clusters were identified as intervention and control based on the intervention (checklist based box system interventions on improving utilization of maternal health service utilization) received	Coded as intervention and control

*Fourth antenatal care visit* – Fourth ANC visits in this study refer to a pregnancy check-up achieved when a pregnant woman receives care at all four appointments. *Knowledge of ANC service* – This variable assessed participants’ understanding of the services required and provided during pregnancy. A composite index of eight variables (detailed in Table 1) was developed and dichotomized using the mean score: those who scored the mean or higher were classified as ‘knowledgeable,’ while those who scored below the mean were classified as ‘not knowledgeable.’ *Knowledge of danger signs of pregnancy* – Participants’ were asked to make a list of potential danger signs that could occur during pregnancy. A composite index of eleven variables (detailed in Table 1) was created and dichotomized using the mean score: those who scored the mean or higher were classified as ‘knowledgeable,’ while those who scored below the mean were classified as ‘not knowledgeable.’ *Gestational age at first ANC* – Participants were asked how far along they were in their pregnancy at their first ANC check-up. This was recoded as ‘less than or equal to 16 weeks’ and ‘greater than 16 weeks of gestation. *Social support*– Social support was measured using the following 14 item questions with ‘yes’ or ‘no’ response categories: ‘gets visits from significant others’, ‘getting useful advises’, ‘discussion on problems’, ‘having care at the time of labor and delivery, ‘feeling loved’, ‘others thankful on them’, ‘getting help on household chores’, ‘help with money at emergency’, ‘help in transportation’, ‘help when sick’, ‘attending community level discussions’, ‘member of any religious cast’, ‘attending public meetings’ and ‘help in case of conflicts’. Following that, a principal component analysis was performed, and a composite index was constructed using the principal components. This was dichotomized using the mean score: those who scored the mean or higher were classified as having ‘high social support,’ while those who scored below the mean were classified as having ‘low social support.’ (Cronbach’s alpha = 0.81).

### Data quality control

Before starting field data collection, training for data collectors and supervisors was provided to create a shared understanding of the study tools. A manual explaining question types and response categories and how to use ODK was produced and distributed to data collectors (BSC-holder midwives) and supervisors (MPH holders). The use of ODK aided in setting questions ‘required’ (to avoid unanswered questions), applying range checks for selected data values, and handling field editing before leaving the respondent.

### Data management and analysis

In addition to the field team, data collection was managed directly from Jimma University, where all study databases were secured with a password-protected access system. Following field data collection, the data were exported from the kobo-tool box and imported into STATA MP Version 15 for analysis.

Data from the trial were analysed using intention to treat analysis. Participants were assigned to clusters based on where they lived at the start of the trial. Because the baseline outcome did not determine the cluster allocation, the difference in difference (DiD) estimator was used to compare the status of the fourth ANC visit between the four contact points (baseline vs. end-line and intervention vs. control) [29]. Similarly, the status of the fourth ANC between the intervention and control arms was compared using the chi-square test of association. Then, to identify factors influencing fourth ANC visit, a bivariate analysis was performed to test the relationship between each independent variables and the outcomes variable, then variables with *p* < 0.25 were included in the multivariable model.

Analysing different levels of factors at a single level using ordinary logistic regression results in loss of power and introduces Type I error. The presence of clustering (individuals are nested in kebeles in this case) also violates the assumption of independence among study participants. As a result, this study employed a mixed effect, multi-level logistic regression model. The multi-level analysis allows for the simultaneous examination of the effects of group and individual level variables on individual-level outcomes while accounting for non-independence of observations within groups. Similarly, this model considers individual probability, which is statistically dependent on the participants’ place of residence. This context dependence was taken into account to obtain accurate regression estimates [30].

The presence of cluster-level variability influencing fourth ANC visit was then tested using the intercept-only model and ICC [31]. In addition, the variation between clusters was measured using the median odds ratio (MOR) [32–36] and proportional change in variance (PCV) [37]. The ICC is used to calculate the proportion of total variance in the outcome attributed to the area level, whereas MOR is used to calculate unexplained cluster heterogeneity [34]. Then, model fitness for the multi-level model was tested by using the log-likelihood ratio (LR) test. Four models were constructed during the analysis: the first model was an empty model that was used to determine how much cluster variation influenced fourth ANC visit. The second model adjusted for individual-level variables, the third model was adjusted for community-level variables and the fourth for both individual and community-level variables. A bivariate analysis was performed first, and variables with *p* < 0.25 were included in the second and third models. Then, variables with a *p* < 0.05 in the second and third models were included in the final model. The *p*-value of < 0.05 was used to define statistical significance, AOR together with 95% CI were used to show the strength of association and level of significance, respectively.

## Result

### Socio-demographic characteristic

#### Baseline

**Fig. 4:**
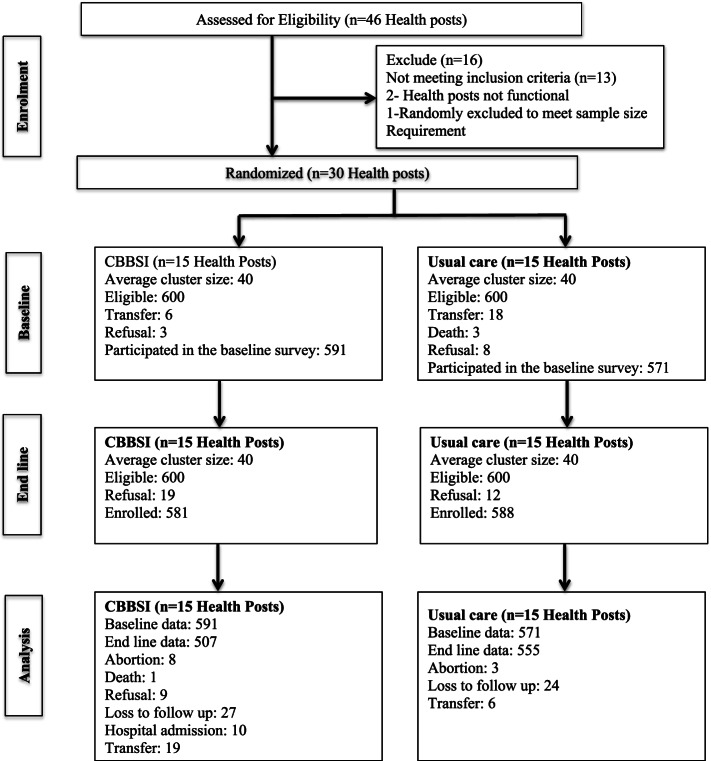
CONSORT flow diagram for cluster trial

Data were collected from 1162 participants, 591 (50.9%) from 15 control and 571 (49.1%) from 15 intervention clusters (Fig. 4). The mean age of study participants was 28.8 years (SD ± 6.1). Most participants were from rural residence 971 (83.6%) and in marital union 1108 (95.4%) (Table 2).

**Table 2:** Socio-demographic characteristics of study participants disaggregated by *baseline and end-line and intervention and control clusters*, East Gojjam Zone, Northwest Ethiopia, January 2019–September 2020 (*n* = 2224)

Variables	Baseline				End line			
	Intervention (***n*** = 571)		Control (***n*** = 591)		Intervention (***n*** = 555)		Control (***n*** = 507)	
	Freq.	%	Freq.	%	Freq.	%	Freq.	%
Age								
15–19	23	4	27	4.6	29	5.2	10	1.9
20–29	339	59.4	252	42.6	345	62.2	352	69.4
30–49	209	36.6	312	52.8	181	32.6	145	28.6
Place of Residence								
Rural	499	87.4	472	79.9	510	91.9	376	74.2
Urban	72	12.6	119	20.1	45	8.1	131	25.8
Educational Status								
No formal Education	171	29.9	160	27.1	169	30.5	115	22.7
Primary (1-8th grade)	343	60.1	381	64.5	330	59.5	293	57.8
Secondary (9-12th grade)	33	5.8	29	4.9	49	8.8	62	12.2
Above 12th Grade	25	4.4	20	3.4	7	1.3	37	7.3
Marital Status								
Single	12	2.1	18	3	10	1.8	6	1.2
Married	548	95.9	560	94.8	530	95.5	490	96.6
Separated	11	1.9	10	1.7	14	2.5	10	1.9
Widowed	0	0	3	0.5	1	0.01	1	0.2
Wealth Index								
Poorest	217	38	195	32.9	207	37.3	199	39.3
Poor	199	34.8	242	40.9	173	31.2	144	28.4
Medium	60	10.5	78	13.3	116	20.9	73	14.4
Rich	74	12.9	68	11.5	46	8.3	64	12.6
Richest	21	3.7	8	1.4	13	2.3	27	5.3
Parity								
One Child	149	26.1	166	28.1	218	39.3	221	43.6
2–4 Children	324	56.7	296	50.1	282	50.8	237	46.7
≥ 5 Children	98	17.2	129	21.8	55	9.9	49	9.7

#### End line

During the end-line survey, data were collected from 1062 participants, 555 (52.3%) from 15 control and 507 (47.7%) from 15 intervention clusters (Fig. 4). The participants in the study had a mean age of 26.9 Years (±5.4) (26.7 and 27.3 years for control and intervention arms, respectively). The majority of participants, 886 (83.4%), were from rural areas. Most of the participants, 1020 (96.1%), were married. The majority of the mothers, 519 (48.9%), had 2–4 children (Table 2).

### Status of antenatal care service utilization

#### Baseline

Most mothers went to health facilities for their first ANC visit, with 555 (92.9%) from the control and 538 (94.2%) from the intervention clusters. Two hundred and seventy-nine (47.2%) mothers in the control and 386 (67.6%) mothers in the intervention arm began ANC within the recommended time frame, before 16 weeks of gestation. Two hundred seventy (45.6%) and 291 (50.9%) mothers from the control and intervention clusters had four ANC visits (Table 3).

**Table 3:** Comparison of ANC visits between two arms of the baseline and end-line participants, Northwest Ethiopia, January 2019–September 2020, *n* = 2224 (Baseline = 1162, End line = 1062)

ANC visits	Baseline		End-line	
	Control clusters (*n* = 591)	Intervention Clusters (*n* = 571)	Control clusters (*n* = 555)	Intervention Clusters (*n* = 507)
No ANC visit	36 (6.1%)	33 (5.8%)	2 (0.4%)	2 (0.4%)
First ANC Visit	555 (92.9%)	538 (94.2%)	553 (99.6%)	505 (99.6%)
Second ANC visit	537 (90.8%)	492 (86.2%)	540 (97.3%)	502 (99.0%)
Third ANC Visit	172 (74.7%)	427 (74.8%)	437 (78.7%)	489 (96.4%)
Fourth ANC visits	270 (45.6%)	291 (50.9%)	297 (53.7%)	432 (85.2%)

#### End line

Almost all mothers, 553 (99.6%) and 505 (99.6%) from the control and intervention clusters, respectively, visit health facilities for the first ANC visit. In terms of the month of initiation, 359 (65.1%) mothers in the control group and 343 (67.9%) mothers in the intervention group began the first ANC before 16 weeks of gestation. During the end-line survey, 297 (53.7%) mothers from the control clusters and 432 (85.2%) mothers from the intervention clusters attended the fourth ANC visit (Table 3).

### Effectiveness of checklist based box system intervention (CBBSI) on improving attendance of the fourth ANC visit

The DiD analysis revealed a statistically significant difference in fourth ANC visit over time between the intervention and control groups (26.1, 95% CI: 18–34%, *p* < 0.0001). Compared to the control group, the proportion of mothers who attended the fourth ANC visit was significantly higher in the intervention clusters (432 (85.2%) vs. 297 (53.7%), *p < 0.0001*) (Table 4).

**Table 4:** Fourth ANC visit and related factors, Northwest Ethiopia, January 2019–September 2020 (*n* = 1062)

Variable	Intervention		Control		*x*^*2*^ test	*P*-value
	Frequency	%	Frequency	%		
Fourth ANC visit						
Yes	432	85.2	297	53.7	124	< 0.0001
No	73	14.5	256	46.3		
Knowledge of ANC service						
Knowledgeable	401	79.1	414	74.6	3.04	0.08
Not-Knowledgeable	106	20.9	114	25.4		
Danger signs of pregnancy						
Knowledgeable	280	50.5	277	54.6	1.86	0.17
Not-Knowledgeable	230	45.4	275	49.5		
Gestational age at First ANC (*n* = 1058)						
≤ 16 weeks	359	65.1	343	67.9	0.98	0.32
> 16 weeks	193	34.9	162	32.9		
Level of social support						
Good	344	67.9	404	72.8	3.11	0.07
Poor	163	32.1	151	27.2		
Influence by significant others						
Yes	60	11.8	36	6.5	9.22	< 0.01
No	447	88.2	519	93.5		
Place of residence						
Urban	131	25.8	45	8.1	60.24	< 0.0001
Rural	376	74.2	510	91.9		
Pregnancy wontedness						
Yes	475	94.2	523	93.7	0.14	0.71
No	32	5.8	32	6.3		
Compassionate and respectful Care (*n* = 953)						
Yes	450	90.7	433	94.7	5.7	0.02
No	46	9.3	24	5.3		

### Factors affecting utilization of fourth antenatal care visit

The results from a mixed effect multi-level logistic regression revealed that, of the individual-level variables, knowledge of the services provided during ANC was significant (model 2), and, of the community-level variables, level of social support and receiving CBBS intervention were significant (model 3). All three variables mentioned above became significant in the final model.

Mothers who were knowledgeable about the services provided during ANC visits were more likely to have the fourth ANC visit (AOR: 2.31, 95% CI:1.65–3.24). Mothers with a high level of social support (AOR:1.47, 95% CI:1.06–2.04) were found to be more likely to attend the fourth antenatal visit than those with a low level of social support. Mothers in the intervention clusters (AOR:5.69, 95% CI:4.14–7.82) were more likely to attend the fourth ANC visit than those in the control clusters (Table 5).

**Table 5:** A multi-level logistic regression analysis of factors affecting fourth ANC visit, Northwest Ethiopia, January 2019–September 2020 (*n* = 1062)

Variables	Model 2 AOR (95%CI)	Model 3 AOR (95%CI)	Model 4 AOR (95%CI)
*Individual-level factors*			
Educational status			
Non-formal	1		
Primary (1-8th grade)	1.05(0.71–1.55)		
Secondary (9-12th grade)	1.50(0.82–2.77)		
Above 12	2.44(0.85–6.97)		
Parity			
one	1		
2-4th	0.95(0.68–1.34)		
≥ 5	0.98(0.55–1.77)		
Knowledge of ANC service			
Knowledgeable	2.27(1.55–3.31)*		2.31(1.65–3.24)***
Not-knowledgeable	1		1
Danger signs of pregnancy			
Knowledgeable	1.39(0.97–1.97)		
Not-knowledgeable	1		
*Community-level factors*			
Place of residence			
Urban		1.31(0.83–2.08)	
Rural		1	
Influence by significant others			
Yes		0.61(0.37–1.02)	
No		1	
MHS free of charge			
Always		6.01(0.56–64.78)	
Most of the time		4.48(0.42–48.18)	
Sometimes		4.09(0.67–45.56)	
Never		1	
Level of social support			
High		1.52(1.09–2.13)	1.47(1.06–2.04)*
Low		1	1
CBBSI Cluster			
Intervention		5.34(3.85–7.40)	5.69(4.14–7.82)***
Control		1	1

Model 1: Intercept only model, *p < 0.05, ***p < 0.0001

This study showed that an increase in the number of deliveries (2–4 deliveries (AOR: 0.95, 95%CI: 0.68–1.34) and five deliveries (AOR: 0.98, 95%CI: 0.55–1.77) was inversely proportional to the numbers attending the fourth ANC visit, but the result was turned insignificant.

The intercept-only model of mixed effect multi-level logistic regression analysis revealed that 18.2% (ICC:18.2%: *p* < 0.0001) of the total variation across communities/clusters is attributable to cluster-level variation. Furthermore, MOR of 2.25, which differs from 1 (no association) in the empty model, confirmed that community-level factors influence fourth ANC visit.

After adjusting for individual-level (Level 1) factors in model 2, the variance attributable to cluster level was reduced to 17.7% (ICC:17.7%: *p* < 0.05), MOR:2.20). Similarly, after adjusting for community level (Level 2) factors in model 3, the variance attributable to cluster level was nearly zero (ICC:0.0009%: *p* < 0.01), MOR:0.73). Finally, after adjusting for both individual and community-level factors in model four, almost 0 % (ICC:0.003%: *p* < 0.05, MOR:0.07) of the variation was attributable to cluster level (Table 6).

**Table 6:** Random intercept model/measure of variation for fourth ANC visit, Northwest Ethiopia, January 2019–September 2020

Measure of Variation	Model 1	*P* value	Model 2	*P* value	Model 3	*P* value	Model 4	*P* value
Variance (SE)	0.73 (0.24)	< 0.0001	0.71 (0.24)	< 0.05	0.003 (0.04)	< 0.01	0.013(0.04)	< 0.05
ICC (%)	18.2		17.7		0.0009		0.003	
MOR	2.25		2.20		0.73		0.07	
PCV	*Reference*		2.7		99		98	
Model Fitness statistics								
Deviance	− 623							
LR test vs Logistic Model (*P*-value)	< 0.0001							

*SE* standard error, *ICC* Intracluster correlation coefficient, *MOR* Median odds ratio, *PCV* Explained variation

Model 1: Intercept only model

Model 2: Adjusted for individual-level variables

Model 3: Adjusted for community-level variables

Model 4: Adjusted for individual and community level variables

Similarly, the result of the final model showed that the variance of both the individual and community-level at model 4 was 0.013. Around 98% of the variation in the fourth ANC visit across the communities was explained by the full model. This showed that a minimal level of variations that were contributed to the fourth ANC visit remained unexplained by the full model.

## Discussion

The implementation of checklist-based box system intervention improved the utilization of the fourth ANC visit in the study area. In this study, community-level factors (cluster and facility/intervention) explained more variation in the fourth ANC visit than individual-level factors. This, in turn, demonstrated that once mothers visited health facilities for ANC follow-up, their attendance at subsequent visits was greatly influenced by factors that operate at a level above the individual level.

This study found that knowledgeable mothers were more likely than their counterparts to receive fourth ANC visit. This finding is consistent with a study conducted in rural Pakistan, which found that knowledgeable mothers are more likely to attend ANC visits [11]. In a study area where more than half of WRA were uneducated and more than 80% of WRA did not have access to at least one of the three common media outlets (radio, television, and newspaper) that the government commonly used to transmit health messages, health-related knowledge will have a significant impact on women’s health-seeking behaviour, including ANC [9]. It is believed that informed mothers understand ANC, the services provided during ANC, and the benefits of receiving care and that this has a positive impact on consecutive attendance at ANC visits.

In this study, a high level of social support was found to be one of the independent predictors of fourth antenatal care visit. Despite Ethiopia’s strong social support structures, the relationship between these structures and individual health behaviour has not been thoroughly investigated. According to studies conducted in Ghana [12] and Canada [13] on the effect of social support structures on prenatal care service utilization, women who belonged to stronger social support structures were more likely to visit health facilities for prenatal care services. Social support structures take advantage of disseminating relevant information, share opinions and experiences about medical care among members, and most importantly to act according to suggestions and general expectations between members of the same group, and thus play an important role in the decision to use health services, including prenatal care services.

Compared to the control arm, most mothers in the intervention clusters attended the fourth ANC visit. This implies that, compared to the routine care, individual-based health educations combined with dropout tracing mechanisms were more effective in attending fourth ANC visits.

In order to implement the intervention, this trial made use of the existing primary health care unit structure and linkages. HEWs, who spent 75% of their time in the field, visited mothers who had missed consecutive ANC visits. The implementation was not reliant on infrastructures such as mobile ownership, network coverage, or power, which is not always practical in Ethiopia.

It is well known that HEWs at health posts have a heavy workload; however, in this trial, mothers who do not follow a smooth transition from the first to the fourth ANC were more targeted than those who attended the recommended visits on time. Most health posts were staffed with two HEWs, a few with three, and rarely with a single HEW, with 75% of their time spent in the field. Implementing CBBS intervention with this proportion would be safe, but in cases where HEWs are displaced from their workplace for various reasons, maintaining a smooth workflow would be critical.

### Strength and limitations

One of the study’s strengths is that it used a double-blind approach. Mothers and outcome assessors were unaware of the intervention. Mothers were unaware that they were in a different treatment group and that they were receiving different interventions. Furthermore, the study relied on the HCP of different groups to deliver the intervention and to collect data. The data collectors were not aware of the intervention, which groups were in the intervention and control groups, or the hypothesis that would be tested. In this process, the study reduce ascertainment bias or detection bias. By comparing baseline and end line results of the outcome of interest between the intervention and control groups, DiD analysis fills a gap that was not considered in simple randomization techniques used. Furthermore, prior to the randomization procedure, health posts/clusters were identified and recruited, as were mothers for the baseline survey. This aided in reducing identification bias. To assess the outcome of interest, the study team used the following question: “How many ANC visits did you have for your last pregnancy?” This was thought to be clear, understandable, and non-leading. The tools were also pretested in similar groups of mothers to ensure their understandability and clarity. Regardless of the aforementioned considerations, there is a possibility of self-reporting bias as long as there is self-reporting. Furthermore, because it is difficult to draw conclusions with the available sample size, the study did not compare maternal and neonatal outcomes between intervention and control groups.

## Conclusion and recommendation

Checklist-based box system intervention was found to be effective in increasing utilization of the fourth ANC visit. Compared to individual-level factors, community/facility level determinants were significantly impacted on fourth ANC visit. As the Ministry of Health is finalizing the implementation period for Health Sector Transformation Plan I and plans for the next 5 years for Health Sector Transformation Plan II, strategies derived from this plan including the reproductive health strategy, should take this trial into account for broader implementation.

## Data Availability

The datasets generated and/or analysed during the current study are not publicly available, because the data set is being used by the organization to generate additional manuscripts by analysing the same data set with different objectives, but are available from the corresponding author on reasonable request (netsanetb2009@gmail.com).

## Abbreviations

ANC: Antenatal care
CBBSI: Checklist based box system intervention
DiD: Difference in Difference
EDHS: Ethiopian Demographic and health survey
HCG: Human chorionic gonadotropin
HCP: Health care provider
HESB: Health education scheduling box
HEW: Health Extension worker
HSTP: Health sector transformation plan
ICC: Intra cluster correlation coefficient
MOR: Median Odds Ratio
ODK: Open data kit
PCV: Proportional change in variance
PRECEDE: Predisposing, Reinforcing, and Enabling Constructs in Educational Diagnosis and Evaluation
PROCEED: Policy, Regulatory, and Organizational Constructs in Educational and Environmental Development
TT: Tetanus Toxoid
WHO: World Health Organization
WRA: Women of Reproductive Age
